# Enhanced Fatigue Limit in Ultrafine-Grained Ferritic–Martensitic Steel

**DOI:** 10.3390/ma16041632

**Published:** 2023-02-15

**Authors:** Marina A. Nikitina, Rinat K. Islamgaliev, Artur V. Ganeev, Aleksandra A. Frik

**Affiliations:** 1 Institute of Physics of Advanced Materials, Ufa University of Science and Technology, 32 Zaki Validi Str., 450076 Ufa, Russia; 2 Laboratory of Multifunctional Materials, Ufa University of Science and Technology, 32 Zaki Validi Str., 450076 Ufa, Russia

**Keywords:** fatigue, UFG, CSL boundaries, cold rolling, requenching

## Abstract

The influence of the ultrafine-grained (UFG) structure on the fatigue endurance limit and the nature of fatigue failure have been studied. It is shown that the formation of the UFG structure containing carbides and the coincidence site lattice relationship (CSL) and twin boundaries leads to an increase in the fatigue endurance limit. To study the mechanisms of fatigue failure, scanning and transmission electron microscopy and X-ray diffraction analysis were used. Studies have shown that the formation of the UFG structure as a result of rolling and subsequent heat treatment above the temperature of the ferrite/austenite phase transition leads to an increase in the fatigue endurance limit by more than 70%, from 475 to 800 MPa, compared to coarse-grained samples. The dynamic aging observed during fatigue tests was more pronounced in materials with a UFG microstructure. The influence of the CSL and twin boundaries on the nature of the fatigue failure of ferritic–martensitic steel is discussed.

## 1. Introduction

9–12% Cr martensitic steels are widely known as common materials for gas turbine components for operation at elevated temperatures and in high-pressure conditions [1,2]. The cyclic response and microstructure sensitivity are the crucial factors influencing the fatigue performance of the turbine steel grades with high chromium content.

It is known that understanding the mechanisms for the enhancement of fatigue endurance is important for fundamental and practical applications [3,4,5,6,7,8,9,10,11]. The characteristics of the fatigue failure of metallic materials are determined by many factors, among which grain boundaries, dispersed particles, and solid-solution hardening play an important role [3,10,11,12,13,14]. In particular, much attention has recently been paid to the development of ultrafine-grained structures (UFG) in metals, which lead to an increase in tensile strength compared to their coarse-grained counterparts. Studies have shown that UFG materials have a higher crack propagation rate in stationary mode [3,15]. According to [3], the grain refinement occurring at severe plastic deformation (SPD) increases the fatigue limit; however, the decrease in the plasticity of SPD samples can negatively affect the nature of fatigue failure. In [16], the authors showed that the formation of UFG structure by equal-channel angular pressing (ECAP) in combination with additional heat treatment above the temperature of the ferrite/austenite phase transition promoted an increase in the fatigue endurance limit by more than 50%, from 475 to 735 MPa. The main features that made it possible to provide such a level of fatigue endurance were a homogeneous UFG structure with an average grain size of 0.8 microns, smaller and uniformly distributed carbide particles of 30–50 nm as well as an increased fraction of boundaries with the CSL boundaries of up to 7%. At the same time, it is well known, that industrial scaling up of SPD methods is very complicated and restrictive in terms of the final component dimensions.

In this regard, the investigation of structural features which significantly increase functional properties using commercial equipment becomes of practical importance. Therefore, the purpose of this work was to investigate the effect of the structure containing an increased fraction of the CSL and twin boundaries on the nature of the fatigue failure of ferritic–martensitic steel subjected to combined processing, including rolling and additional heat treatment above the ferrite/austenite phase transition temperature.

It is known that twin boundaries can be strong barriers to dislocation motion preventing the dislocation glide [17] that usually accompanies the initiation and growth of a crack. As a result of twinning, a significant increase in strength is observed in a material [18]. The novelty of this paper was to show that the formation of the structure containing numerous CSL and twin boundaries will suppress the crack growth from splitting the crack into several microcracks which can, for example, lead to the improvement in the fatigue properties of ferritic–martensitic steel.

## 2. Materials and Methods

Ferritic–martensitic steel 12Cr–2W–2Ni–0.5Mo, which is frequently used in power engineering up to a temperature of 600 °C, was investigated. The chemical composition of the steel is presented in Table 1.

**Figure 1:**
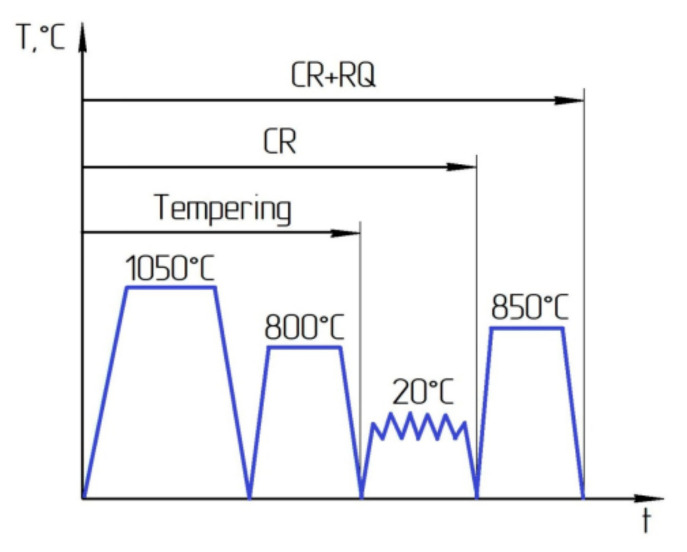
Schematic illustration for heat treatment processes applied to the steel samples.

The standard heat treatment procedure for steels with a content of 9–12% Cr is quenching, i.e., heating above the temperature of phase transitions with rapid cooling [16]. The subsequent high-temperature tempering relieves internal stresses and leads to greater structure equilibrium. For 12Cr–2W–2Ni–0.5Mo steel, the standard heat treatment is quenching from a temperature of 1050 °C into oil and tempering at 550–710 °C, followed by air cooling. Because the material undergoes large plastic deformation, tempering at a temperature of 800 °C (Figure 1) before cold rolling has been used to obtain complete defect-free UFG samples with a UTS of 950 MPa and ductility of 7% [18].

**Figure 2:**
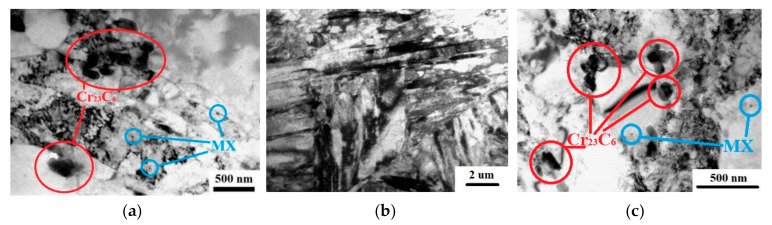
Microstructure of steel: (**a**) initial hot-rolled state, (**b**) oil quenching, and (**c**) tempering. Bright-field TEM images.

The microstructure of the steel in the initial as-received hot-rolled state is represented by ferritic grains, where the clusters of the carbide particles could be seen along the boundaries (Figure 2a). When the samples were annealed in the temperature range of 1040–1050 °C, almost complete dissolution of carbides occurs, and with further oil quenching, martensite is formed (Figure 2b). Subsequent tempering at a temperature of 800 °C leads to the decomposition of martensite (Figure 2c) and the precipitation of the M_23_C_6_ carbides (50–170 nm in size) and MX carbonitrides (30–50 nm in size) [16].

Plates with dimensions of 28 × 14.5 × 85 mm were made from steel bars with a diameter of 40 mm after tempering. The samples were rolled at room temperature to a reduction of 50% by cold rolling (CR) on a laboratory rolling mill at a speed of 1.5 m/min and a reduction of 0.5 mm per pass. The cold rolled samples were subjected to heat treatment above the temperature of the ferrite–austenite phase transition (850 °C, with exposure for 30 min), followed by oil quenching (CR + requenching) (Figure 1).

For X-ray and transmission electron microscope (TEM) analysis, disks with a diameter of 3 mm were cut near the fracture surface. The first disk was cut at a distance of 0.3 mm from the fracture surface, followed by a step of 0.5 mm. To calculate the lattice parameter and the sizes of the coherent scattering domains, we used diffraction patterns obtained with a Rigaku Ultima IV diffractometer employing the Bragg–Brentano focusing method (goniometer geometry). The microstructure was characterized using a JEM-2100 transmission electron microscope. Thin foils for TEM investigations were punched from the thin sections of the samples. They were then mechanically ground to a thickness of 0.15 mm, and finally double-jet electropolished to perforation using an electrolyte based on n-butyl alcohol. To study the fracture surface, a JSM-6390 scanning electron microscope (SEM) with an accelerating voltage of 30 kV was used [19]. Samples that failed during fatigue tests at the level of the fatigue endurance limit were used for investigations of fracture surfaces by SEM. Additionally, these samples were cut by electrospark erosion at a distance of 0.5 mm, 1 mm, and 1.5 mm from the fracture surface in a normal plane to gauge the direction for TEM and X-ray investigations.

A Scanning electron microscope, FEG-SEM TESCAN MIRA 3 LMH, operating at 20 kV was used for electron backscattered diffraction (EBSD) investigations with the scanning step of 100 nm. For the analysis of the Kikuchi lines, CHANNEL 5 software from Oxford Instruments was used. The error in determining the orientation of the crystal lattice was not more than 0.6°. The low-angle boundaries between local volumes were plotted on orientation maps with the misorientation angles ranging between 2–15°, while high-angle boundaries were considered at the misorientation angles over or equal to 15°.

**Figure 3:**
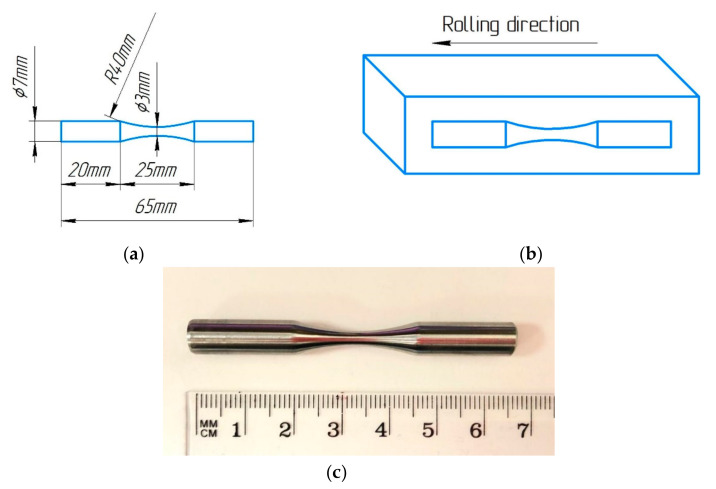
(**a**) Drawing of the fatigue test sample, (**b**) scheme of cutting samples for fatigue testing from CR specimens, and (**c**) photo of specimen for fatigue test.

To determine the fatigue endurance limit, cylindrical samples with a gauge diameter of 3 mm were used (Figure 3). The fatigue tests were carried out in a symmetrical loading cycle with a frequency of 50 Hz using 12 samples per condition and a stress ratio of R= −1.

The investigated steel is frequently used as a material for the manufacture of blades for ground gas turbine engines. During operation, the blades experience loads associated with rotation and bending. Therefore, to study the fatigue properties, the rotating bending fatigue test was chosen with a ratio of R = −1.

In works [20,21], the influence of various stress ratios R (ranging from R = −1 to R = 0.5) on the fatigue endurance limit was studied and it was shown that the maximum value of the fatigue endurance limit was observed at R = −1, and the minimum at R = 0.5.

When loading according to the rotation bending (R = −1), inhomogeneous stresses are developed in the sample and the maximum stresses occur only on the surface of the sample; therefore, in the center of the sample, stresses are equal to zero.

For comparison, a uniform stress distribution is created in the specimen tested in the loading cycle with a ratio equal to R = 0.1. In this case, the entire cross-section of the sample is involved in the deformation process, not just the surface layers as in the case of rotational bending.

Therefore, the propagation of the cracks through the center of the sample is slower in the case of R = −1, and the fatigue endurance limit can be higher when compared to R = 0.1.

## 3. Results

**Figure 4:**
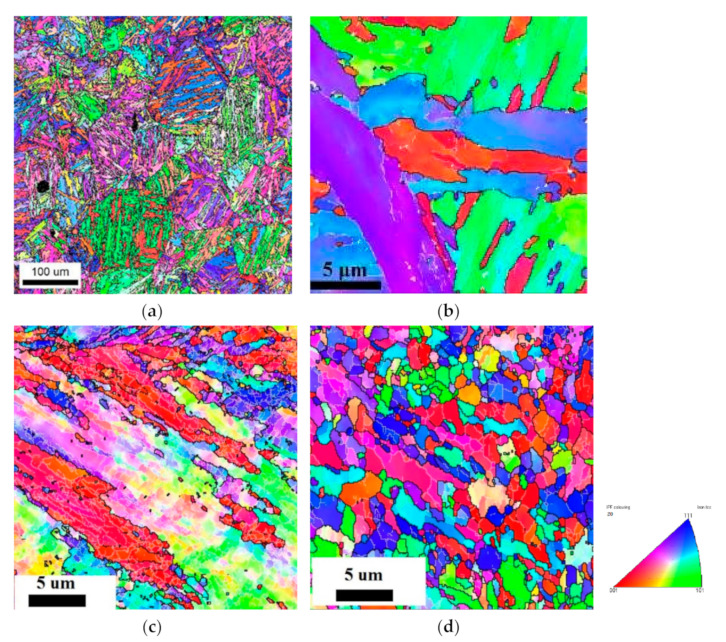
EBSD maps after (**a**,**b**) tempering; (**c**) CR; and (**d**) CR + requenching.

The microstructure of the samples after tempering, CR, and CR + requenching was investigated using electron backscattered diffraction (Figure 4, Table 2). After tempering, the coarse-grained structure with a mean grain size of 150 μm was observed. Plates with an average width of 5 μm were visible inside the grains. (Figure 4b, Table 2). Numerous plates could be seen inside one grain (Figure 4a,b). The application of CR led to the appearance of elongated grains (Figure 4c) and increased the fraction of the high-angle boundaries (HAB) to 44%, while the fraction of boundaries with the coincidence site lattice (CSL) relationships (the CSL boundaries) and twin boundaries (Σ3, Σ11) significantly decreased (Table 2). For the CR + requenched samples, a more uniform microstructure with a mean grain size of 0.9 μm was observed (Figure 4d). The volume fraction of the HAB slightly decreased to 34%, but at the same time, there was an increase in the fraction of the CSL and twin boundaries.

**Figure 5:**
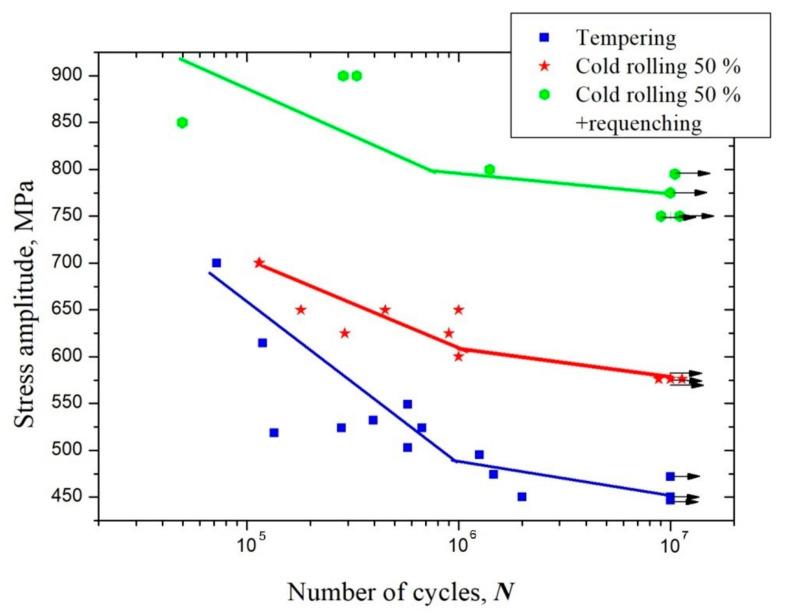
S–N diagram of the behavior of the ferritic–martensitic steel (include the grade here).

Figure 5 shows the S–N diagram of the samples after tempering, CR, and CR + requenching. The fatigue endurance limit based on 10^7^ cycles increases from 450 MPa after tempering to 590 MPa after CR. The application of the heat treatment above the ferrite/austenite phase transition temperature allows for an additional increase in the fatigue endurance limit up to 800 MPa, which is 70% higher than the limit achieved after the tempering.

It is known that twin boundaries act as barriers to dislocation movement [17] that usually accompany the initiation and growth of a crack. From the microstructure of the samples subjected to CR + requenching (Table 2), numerous microtwins can be observed, which, in this instance, suppresses the overall crack growth as it is splitting into several microcracks. It has recently been demonstrated that the formation of the ultrafine-grained structure and microtwins by using equal-channel angular pressing leads to the enhancement of the fatigue endurance limit [16]. This work demonstrated that the formation of microtwins can enhance fatigue life in the structures containing elongated grains in a material subjected to CR (Table 2).

**Figure 6:**
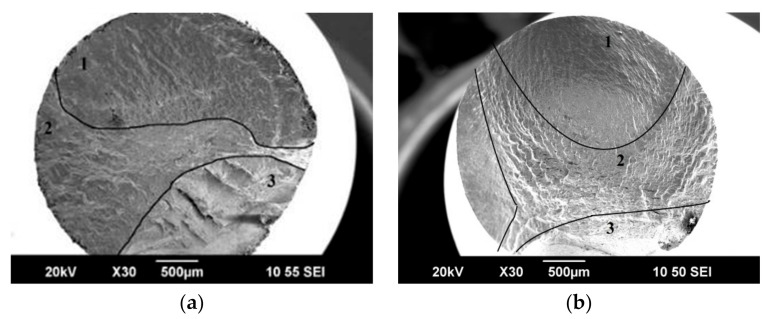
The fracture surface: (**a**) after CR 50% and (**b**) after CR + requenching. The surface of higher magnification is shown in Figure 6 and Figure 7. 1—crack initiation zone, 2—zone of the crack propagation, 3—rupture zone. SEM images.

**Figure 7:**
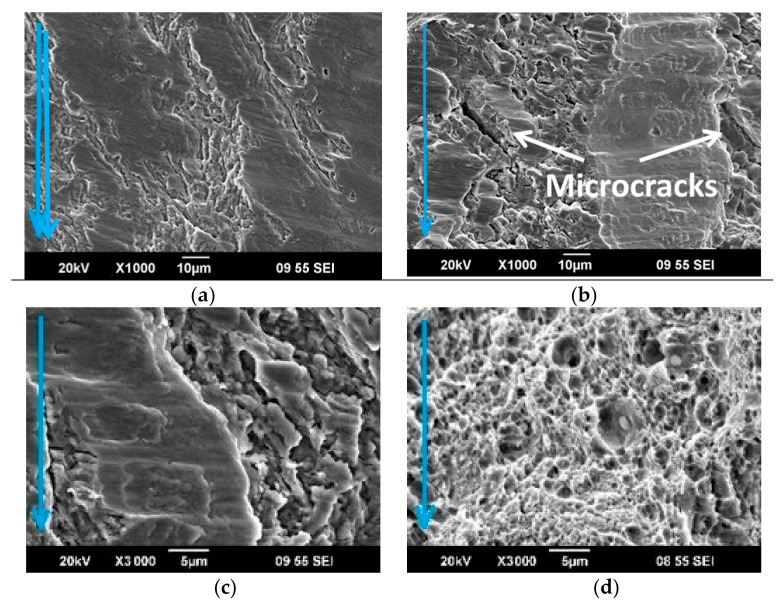
The fracture surface after CR in the sections: (**a**) zone 1; (**b**,**c**) zone 2; and (**d**) zone 3. SEM images. The blue arrows indicate the direction of crack propagation.

To analyze the nature of fatigue failure, a fractographic analysis of fracture surface relief was carried out. At the fracture surface of both CR and CR + requenched samples, a smooth zone depicting the fatigue crack propagation and a rupture zone is clearly visible (Figure 6). Figure 6a shows the rupture of the sample after CR at a load of 750 MPa after 1.5 × 10^6^ cycles with the fatigue crack inclined to nucleate at the surface of the material. The TEM analysis showed that zone 1 (Figure 6a, zone 1) is characterized by scars located in the direction of crack propagation, and the microstructure of this zone reveals a 0.9 mm wide plateau with fatigue grooves, the width of which reaches 30 microns (Figure 7a). The zone of crack propagation is characterized by the presence of facets and grooves. At larger magnifications (i.e., ×1000), an intergranular fracture is observed and secondary cracks are visible (Figure 7b). The failure in the rupture zone occurred visibly with the formation of pits. The sizes of the pits were from 1 to 5 microns, inside which particles of 0.5–2 microns were observed, and the width of the zone was 0.5 mm (Figure 7d).

**Figure 8:**
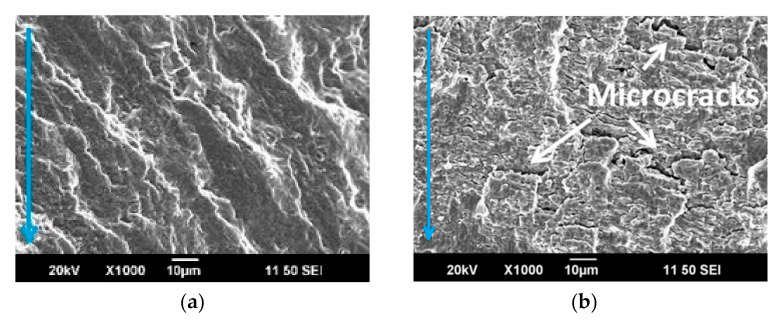

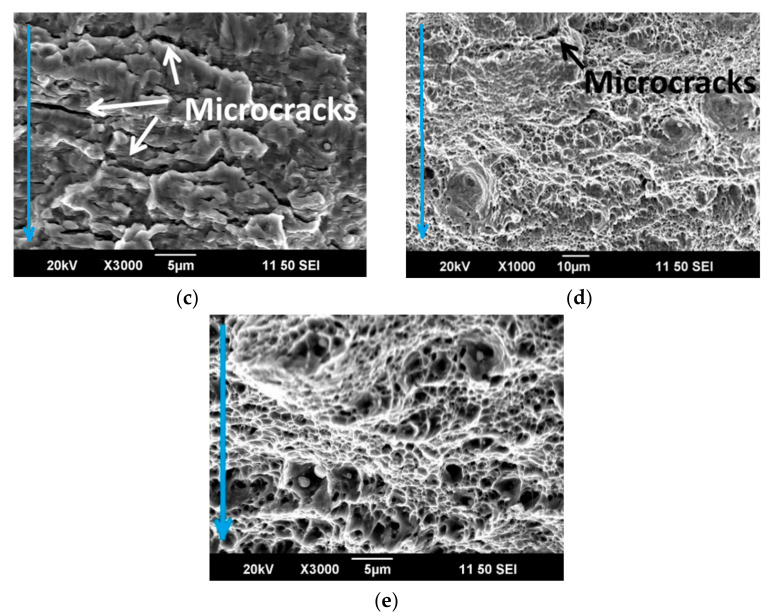
The fracture surface after CR + requenching in the sections: (**a**) zone 1; (**b**,**c**) zone 2; (**d**,**e**) zone 3. SEM images. The blue arrows indicate the direction of crack propagation.

Fractographic studies of the fracture surface of the CR + requenched samples showed that the crack initiation starts at the surface defects. The crack initiation zone 1 (Figure 6b, zone 1) is characterized by scars located in the direction of the crack propagation (Figure 8a). The surface in the zone of the crack propagation is characterized by clearage facets and fatigue striations. At larger magnifications (×1000), a dimpled appearance which is typical for ductile fracture was observed. Numerous secondary cracks developed perpendicular to the direction of crack propagation, which is a result of the local stress relaxation (Figure 8b–d). The rupture occurred visibly with the formation of pits. The size of the pits ranged from 1 to 15 microns, inside which particles of 0.5–2 microns were observed (Figure 8e).

**Figure 9:**
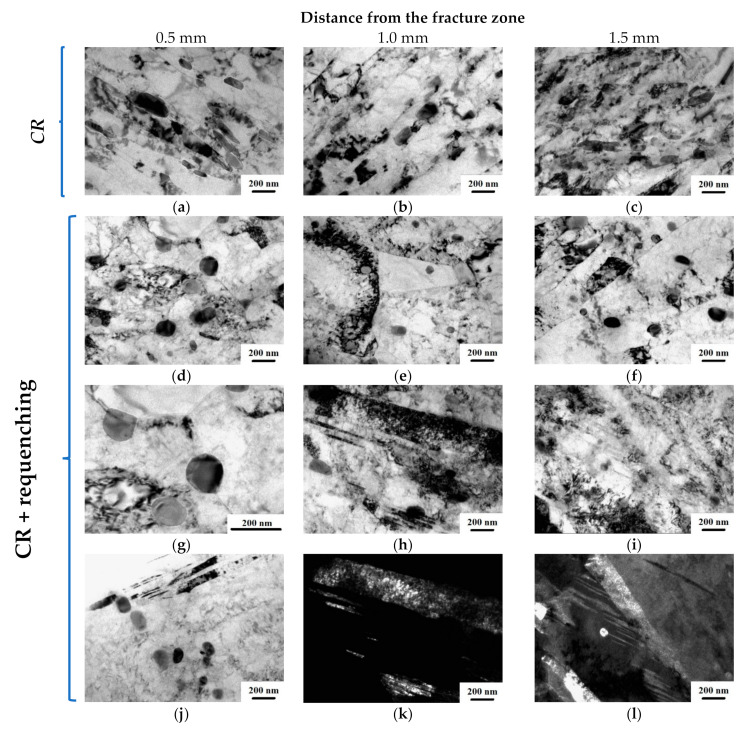
Microstructure of the CR and CR + requenching samples after fatigue tests at a distance of 0.5, 1.0, and 1.5 mm from the fracture zone. TEM images: (**a**–**j**) bright field, (**k**,**l**) dark field.

Figure 9 shows the microstructure of the steel after fatigue tests. According to TEM investigations of the CR samples, at a distance of 0.5 mm from the fracture zone, a nonuniform distribution of carbide particles remains in the microstructure. The strip structure, formed as a result of the rolling, is preserved. With increasing distance from the fracture zone, no significant differences in the microstructure were revealed. The microstructure also shows bands with high dislocation density and nonuniform distribution of carbides and carbonitrides.

**Figure 10:**
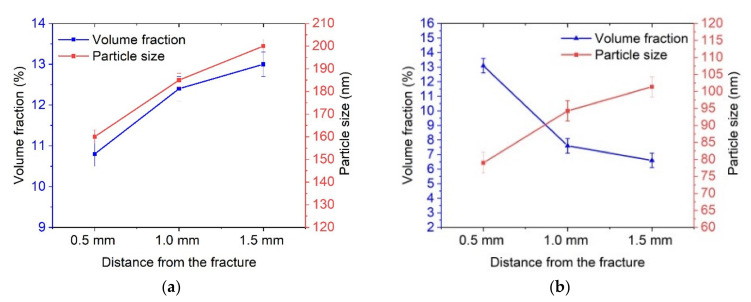

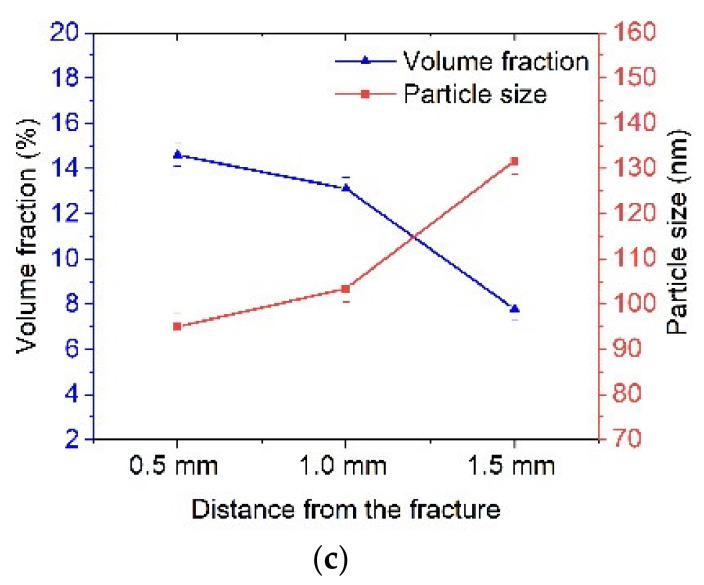
Changes in the structural parameters of the steel after fatigue tests depending on the distance from the fracture zone: (**a**) tempered sample, (**b**) CR sample, and (**c**) CR + requenching sample.

As can be seen from Figure 10, the volume fraction of particles in the fracture zone increased from 6–8% up to 12%, while smaller particles in the order of 80 nm were also observed.

TEM analysis of the structure evolution after fatigue tests of CR + requenched samples showed that an increase in the volume fraction of carbides is also observed directly near the fracture zone and caused by the activation of diffusion processes during rolling (Figure 9d–l). However, there is a uniform distribution of particles belonging to Cr_23_C_6_ both in the grain body and along the boundaries. Figure 9h–l show that the grains have twin boundaries with an average width of 273 ± 23 nm and contain a high dislocation density. As we move away from the fracture zone, the volume fraction of carbides decreases to 8% (Figure 10c).

**Figure 11:**
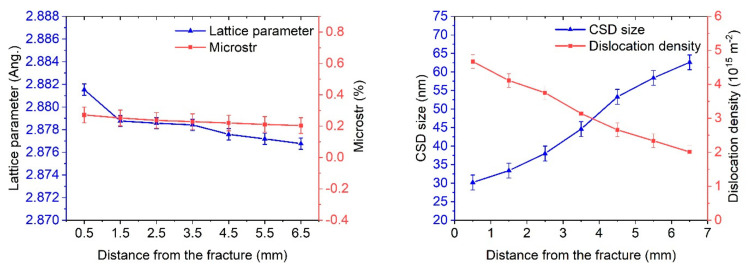
X-ray diffraction analysis after fatigue test of CR + requenching samples.

Dynamic aging occurs in the fracture zone of the CR + requenched samples. As a result, the volume fraction of carbide particles rises and the lattice parameter increases (Figure 11). This observation was confirmed by TEM investigations (Figure 9).

It is known that steel alloyed with tungsten, molybdenum, nickel, and chromium is characterized by increased strength due to dispersion hardening [19,22,23]. For precision analysis of the precipitates formed in the ferritic–martensitic steel, the transmission X-ray diffraction (XRD) technique using the Rigaku Ultima IV diffractometer was used [19].

Table 3 provides information on all carbides and their calculated volume fractions. From the data in Table 3, it can be seen that during the CR + requenching, a significant increase in the volume fraction of particles occurred.

According to the XRD, an increase in the lattice parameter in the fracture zone was observed in the CR + requenched samples. This was evidenced by the dissolution of carbides in the fracture zone. It was also found that there was an increase in the dislocation density directly in the fracture zone by 1.5–2 times; however, as we move away from the fracture zone, there is a gradual recovery in the structure (Table 4).

## 4. Discussion

It was shown that the use of CR for the grain refinement of tempered samples led to an increase in the fatigue endurance limit of up to 590 MPa (Figure 5). The increase in the fatigue limit in the CR samples was obviously associated with both grain refinement and dispersion hardening. It should be noted that the role of grain refinement and dispersion hardening in the enhancement of strength and fatigue endurance limit in various materials is well established [17,24,25,26]. In the present study, the possibility of an additional increase in the fatigue endurance limits up to 800 MPa due to additional heat treatment in the form of quenching from a temperature above the ferrite–austenite phase transition temperature has been shown for the first time.

The following structural changes in the CR + requenched samples compared to CR samples can be noted, which may be responsible for the additional fatigue endurance limit enhancement.

First, a more uniform microstructure was observed with equiaxed grains of 0.9 microns in size (Figure 4d) compared to the microstructure of the CR samples, which are characterized by elongated grains of 10–20 microns long (Figure 4c).

Secondly, additional requenching led to an increase in the fraction of carbides and a more uniform distribution of carbides along the boundaries and in the grain bodies (Table 3, Figure 9d–f).

Third, in the microstructure of the CR + requenched samples, the fraction of the CSL and twin boundaries increased significantly (Table 2).

As a result, the enhanced fatigue endurance limit of the CR + requenched samples compared to the CR samples may be due to several factors: additional grain refinement, increased fraction and more uniform distribution of carbides, and an enhanced fraction of the CSL and twin boundaries.

From the point of view of the contribution of these structural changes to fatigue enhancement in the CR + requenched samples, the following can be said.

The grain refinement in the CR + requenched samples is not sufficiently significant (Figure 4, Table 2) to change the fatigue endurance limit from 590 MPa to 800 MPa.

The total volume fraction of carbides changed from 2.37 in the CR samples compared to 3.17 in the CR + requenched samples (Table 2), which may have contributed to the enhancement of the fatigue endurance limit but not to the same extent.

The fraction of the CSL and twin boundaries in the CR + requenched samples increased by five times compared to the CR samples, which may indicate that the enhanced fatigue endurance limit in the CR + requenched samples compared to the CR samples was mainly due to the additional formation of the CSL and twin boundaries as a result of requenching from a temperature above the ferrite–austenite phase transition temperature. Taking into account the small width of nanotwins of 25 nm in the CR + requenched samples (Figure 9h–l), this assumption regarding the main contribution of the CSL and twin boundaries to the additional enhancement of the fatigue endurance limit may be realistic. In this case, the CSL and twin boundaries may have acted as strong barriers to the dislocation glide that accompanies crack growth. As a result, the crack splits into several microcracks increasing the fatigue endurance limit.

## 5. Conclusions

The application of cold rolling to tempered ferritic–martensitic steel leads to an increase in the fatigue endurance limit from 450 MPa to 590 MPa due to grain refinement and dispersion hardening. Additional requenching made it possible to significantly increase the fatigue endurance limit up to 800 MPa.

The main structural features providing such an increase in the fatigue endurance limit of the CR + requenched samples were a homogeneous UFG structure with an average grain size of 0.9 microns, a uniform distribution of carbides with a size of 30–50 nm as well as an increased fraction of the CSL and twin boundaries.

Changes in the damaging mechanisms after fatigue tests of the CR + requenched samples compared to the CR samples demonstrated the appearance of numerous secondary cracks perpendicular to the direction of crack propagation. In this case, the CSL and twin boundaries are capable of accumulating dislocations that accompany crack growth. As a result, the crack splits into several microcracks increasing the fatigue endurance limit.

## Figures and Tables

**Table 1:** Chemical composition of steel, wt.%.

C	Si	Mn	Ni	S	P	Cr	Mo	W	V
0.1–0.16	up to 0.6	up to 0.6	1.5–1.8	up to 0.03	0.03	10.5–12	0.35–0.5	1.6–2	0.18–0.3

**Table 2:** Parameters of the microstructure from the EBSD investigation.

Condition	Average Grain Size, Microns	Width of Plates, Microns	Fraction of HAB, %	Fraction of CSL Boundaries, %	Fraction ∑3, %	Fraction ∑11, %
Tempering	150 ± 5	5.8 ± 0.2	39 ± 1	11.5 ± 0.3	8.9 ± 0.2	1.8 ± 0.1
CR	-	0.6 ± 0.1	44 ± 0.3	1.4 ± 0.1	0.7 ± 0.1	0.5 ± 0.01
CR + requenching	0.9 ± 0.1	-	34 ± 0.3	7.5 ± 0.2	5.8 ± 0.2	1.1 ± 0.1

**Table 3:** Microstructure parameters from X-ray diffraction analysis. Measurement errors related to the last significant digit are indicated in parentheses.

Condition	*a*, ang.	*D_cp_*, nm	ε, %	ρ, 10^15^ m^−2^	Volume Fraction of Carbides f, %			
					Cr_23_C_6_	Fe_3_C	Fe_3_W_3_C	Total
Tempering	2.87824 (8)	138 (12)	0.12	1.23	0.82	0.46	0.62	1.9
CR	2.87985 (8)	93 (5)	0.23	2.58	1.21	0.45	0.71	2.37
CR + requenching	2.88187 (7)	96 (5)	0.29	3.05	1.72	0.56	0.89	3.17

**Table 4:** XRD and TEM after the fatigue tests of the CR + requenching samples.

State	Lattice Parameter, Ang.	CSD Size, nm	Microstr, %	Disl. dens., 10^15^ m^−2^
0.5 mm fracture	2.881534	30.2	0.271	4.67
1.5 mm fracture	2.878757	33.4	0.252	4.11
2.5 mm fracture	2.878563	38.0	0.237	3.75
3.5 mm fracture	2.878415	44.6	0.229	3.14
4.5 mm fracture	2.877584	53.3	0.220	2.66
5.5 mm fracture	2.877188	58.4	0.211	2.34
6.5 mm fracture	2.876761	62.6	0.204	2.01

## Data Availability

Not applicable.

## Nomenclature

UFG	ultrafine-grained structure
CSL	coincidence site lattice boundaries
SPD	severe plastic deformation
ECAP	equal-channel angular pressing
CR	cold rolling
TEM	transmission electron microscope
SEM	scanning electron microscope
EBSD	electron backscatter diffraction
HAB	high-angle boundaries
XRD	X-ray diffraction
